# Rates of Chronic Medical Conditions in 1991 Gulf War Veterans Compared to the General Population

**DOI:** 10.3390/ijerph16060949

**Published:** 2019-03-16

**Authors:** Clara G. Zundel, Maxine H. Krengel, Timothy Heeren, Megan K. Yee, Claudia M. Grasso, Patricia A. Janulewicz Lloyd, Steven S. Coughlin, Kimberly Sullivan

**Affiliations:** 1 Research Service, VA Boston Healthcare System, Boston, MA 02130, USA; cgzundel@bu.edu (C.G.Z.); mhk@bu.edu (M.H.K.); meganyee@bu.edu (M.K.Y.); claudia.grasso@va.gov (C.M.G.); 2 Division of Graduate Medical Sciences, Behavioral Neuroscience, Boston University School of Medicine, Boston, MA 02118, USA; 3 Department of Neurology, Boston University School of Medicine, Boston, MA 02118, USA; 4 Department of Biostatistics, Boston University School of Public Health, Boston, MA 02118, USA; tch@bu.edu; 5 Department of Environmental Health, Boston University School of Public Health, Boston, MA 02118, USA; paj@bu.edu; 6 Department of Population Health Sciences, Medical College of Georgia, Augusta University, 1120 15th Street, Augusta, GA 30912, USA; scoughlin@augusta.edu

**Keywords:** Gulf War, veterans, Gulf War Illness, Fort Devens Cohort, NHANES, chronic conditions, pyridostigmine bromide, chemical weapons

## Abstract

Prevalence of nine chronic medical conditions in the population-based Ft. Devens Cohort (FDC) of GW veterans were compared with the population-based 2013–2014 National Health and Nutrition Examination Survey (NHANES) cohort. Excess prevalence was calculated as the difference in prevalence estimates from the Ft. Devens and NHANES cohorts; and confidence intervals and *p*-values are based on the standard errors for the two prevalence estimates. FDC males were at increased risk for reporting seven chronic medical conditions compared with NHANES males. FDC females were at decreased risk for high blood pressure and increased risk for diabetes when compared with NHANES females. FDC veterans reporting war-related chemical weapons exposure showed higher risk of high blood pressure; diabetes; arthritis and chronic bronchitis while those reporting taking anti-nerve gas pills had increased risk of heart attack and diabetes. GW veterans are at higher risk of chronic conditions than the general population and these risks are associated with self-reported toxicant exposures.

## 1. Introduction

From August 1990 to June 1991, nearly 700,000 U.S. troops were deployed to the Persian Gulf to serve in Operations Desert Shield and Desert Storm. Shortly after returning, many Gulf War (GW) veterans began reporting a variety of symptoms in multiple body systems that medical professionals could not diagnose. This array of symptoms was termed “Gulf War Illness” (GWI) or “Chronic Multisymptom Illness” (CMI) [1,2]. Researchers have found that this illness continues to affect about a third of all GW veterans [3]. A recent meta-analysis of health symptoms experienced by GW veterans conducted by our research team found that the most commonly reported concerns were fatigue, pain, cognitive and mood problems, skin rash, gastrointestinal and respiratory conditions [4]. This symptom complex was consistent with what was found in a Kansas cohort, which was used to determine one of the two most widely used case criteria [1].

While the exact etiology and pathobiology of these symptoms remains undetermined, research clearly suggests that neurotoxicant exposures in theater contributed to the disorder [5,6,7,8]. GW exposures that have been implicated in the etiology of GWI include low-level sarin chemical warfare agent, pesticides and prophylactic anti-nerve agent pills (pyridostigmine bromide; PB) [5,8]. These exposures can result in both immediate and delayed health effects including chronic cognitive and mood problems and have recently been termed ‘toxic wounds’ to describe this cause-effect relationship [5,7].

Correspondingly, GW veterans have shown increased prevalence of chronic multisymptom illnesses, including GWI, fibromyalgia [1,3,9,10], chronic fatigue syndrome (CFS) [1,3,5,8,9,10,11,12,13,14] irritable bowel syndrome (IBS) [9,13], and functional gastric disorders, for which they can receive presumption for health care service at VA hospitals. GW veterans are also more likely to develop chronic neurological disorders, which may or may not be associated with GWI but have been associated with exposures during the war, including amyotrophic lateral sclerosis (ALS) [15,16,17], brain cancer [3,5,18,19,20], repeated seizures [1,3,5,9,21,22], neuralgias and neuritis [3,5,9,22] and chronic migraine headaches [1,3,5,11,12,13,22,23].

For non-neurological disorders, there have been reports of higher rates of arthritis [1,9,11,12,13,14,22], various lung diseases [1,9,12,13,14,21,22,24], eye or vision problems [1,22], high blood pressure [1,9,10,12,13,14,22], and heart disease [1,9,11,14,21,22] in GW veterans relative to other veteran control groups (GW vs. GW-era veterans, deployed vs. non-deployed veterans, GWI vs. non-GWI veterans). We are unaware of any studies to date in which GW veterans have been compared with the general population on chronic health outcomes. Therefore, it remains unknown whether GW veterans are showing more age-related chronic conditions than the general population and whether or not toxicant exposures are associated with these conditions. There is precedent for this type of toxicant-induced disorder in veterans from other wars. For instance, many Vietnam veterans developed chronic conditions of high blood pressure, diabetes and many types of cancers which the National Academy of Sciences’ (NAS) Institute of Medicine (IOM) concluded were related to remote exposure to Agent Orange [25]. The conditions mentioned above related to GW deployment are also of particular relevance, as one of the most commonly used GWI criteria, the Kansas criteria, excludes chronic medical conditions such as heart disease and diabetes. It is, therefore, necessary to compare the rates of various medical conditions in GW veterans to the general population using a well-established national data set.

One of the earliest and the longest-running studies of GW veterans’ health symptoms was conducted with the Ft. Devens, MA Cohort (FDC). This cohort was surveyed in regard to their health symptom reporting and provided important information regarding the rates and types of current health patterns [26,27]. Results of initial studies from the early 1990s revealed that joint pain, headaches, memory and attention difficulties, skin rash, gastrointestinal difficulties and sleep problems were the most commonly reported chronic symptoms 24 months post-deployment. Early FDC studies also showed that women and those reporting taking 20 or more PB pills were more likely to meet criteria for GWI [26]. In the most recent survey of this cohort it was found that individuals are still experiencing health symptoms at high rates [28]. What was not initially clear was whether or not these rates were higher than those in the general public.

Epidemiologic studies of health symptoms have compared rates of cohort samples to the National Health and Nutrition Examination Survey (NHANES) data set. This is a program of studies created by the Centers of Disease Control and Prevention (CDC) to assess the health and nutritional status of adults in the United States. The NHANES surveys began in the early 1960s and continue to this day.

In the current study, we build upon, extend and update prior work by comparing the rates of chronic conditions in GW veterans to that of the general population. Specifically, we compared the FDC survey data to the general population (2013–2014 NHANES), to compare rates of self-reported doctor diagnosed chronic medical conditions between cohorts. We also investigated the health status of GW veterans from the FDC survey of 2013–2014 as it relates to self-reported neurotoxicant exposures. We hypothesized that GW veterans would have higher rates of chronic conditions than the general population. We also hypothesized that GW veterans who reported neurotoxicant exposures would have higher rates of chronic conditions than GW veterans who did not report neurotoxicant exposures, similar to other deployment-related toxicant exposed veteran groups.

## 2. Materials and Methods

### 2.1. Participants

Comparison data for this study came from two different population-based cohorts. The first cohort included the Ft. Devens, MA Cohort of GW veterans (FDC), and the second cohort was the NHANES dataset. The FDC is one of the few longitudinal cohorts of GW veterans and is the longest running cohort of GW veterans. The most recent Time 5 resurvey began in 2013 and 448 participants who had data for at least one medical condition responded to the FDC Reunion Survey (47 women). Data for the comparison group (*n* = 2949) was obtained from the NHANES survey for the year 2013–2014, restricted to the age range of the FDC respondents. For the current analyses, veterans were excluded from the NHANES sample.

### 2.2. Self-Report of Medical Conditions

The FDC veterans were asked to self-report if a doctor had ever diagnosed them with 9 chronic medical conditions: high blood pressure (HBP), high cholesterol, heart attack, diabetes, stroke, cancer, asthma, arthritis, and chronic bronchitis. Responses were recorded dichotomously (yes/no). NHANES participants also self-reported whether or not a doctor had ever diagnosed them with these conditions (yes/no) and these data were compared with the FDC survey data.

### 2.3. Self-Report of Exposures

FDC veterans were asked during this resurvey to self-report if they were ever exposed to chemical or biological warfare (CBW) agents or pyridostigmine bromide (PB) anti-nerve agent pills. CBW was used as a proxy for chemical weapons (sarin/cyclosarin) exposure.

### 2.4. Statistical Analysis

Since the FDC and NHANES used different sampling strategies, prevalence estimates and their standard errors were calculated separately for each cohort. NHANES employed a complex survey design and prevalence was estimated using SAS survey procedures to account for weighting and clustering in estimating both parameters estimates and standard errors. The FDC was analyzed as a random sample from the Ft. Devens population. Excess prevalence was calculated as the difference in prevalence estimates from the Ft. Devens and NHANES cohorts, and confidence intervals and *p*-values are based on the standard errors for the two prevalence estimates. Odds ratios and test-based confidence intervals were calculated from the prevalence estimates and standard errors from the two cohorts, similar to what was done in Yun et al. 2006 [29].

Comparisons were made separately for men and women to assess for gender- and age-specific associations. For the age-specific associations, we compared three different age groupings: 40s, 50s, and 60s+ by cohort. Reflecting the Ft. Devens population, the FDC sample was largely white (94%), with high school education or higher (99%). To account for demographic differences between cohorts, we restricted both cohorts to White/Caucasian individuals with a high school or above education. After restriction, there were some demographic differences between cohorts on age (Ft. Devens women were younger) and education (Ft. Devens men were more likely to have other training or some college), and analyses adjusted for these demographic differences by modifying the NHANES weights to match the age and education distribution, for men and women separately, of the Ft. Devens cohort. After restriction and weighting (now listed as restricted analyses), there were no significant differences in current smoking between the two cohorts.

Chi-square tests of independence were used to examine the association between gender and medical conditions, as well as the association between exposures and medical conditions, and to calculate odds ratios in the FDC. Multinomial logistic regressions were run to obtain the adjusted ORs, when applicable. For all analyses, two-sided *p* < 0.05 was considered significant, and no adjustments were made for multiple comparisons.

*Data Availability Statement:* Analyses were performed using raw data that are only available within the US Department of Veterans Affairs firewall in a secure research environment. VA privacy and data security policies and regulatory constraints provide that only aggregate summary data may be removed from the VA for publication. The authors have provided detailed results of these analyses in the paper. These restrictions are in place in order to maintain patient privacy and confidentiality. Access to these data can be granted to persons who are not an employee of the VA; however, there is an official protocol that must be followed for doing so. The authors invite those wishing to access the raw data that were used for this analysis to contact Maxine Krengel (Maxine.Krengel@va.gov) to discuss the details of the VA data access approval process.

## 3. Results

### 3.1. Demographics

The mean age of the FDC was 53.93 years. The mean age of the NHANES cohort was 55.86 years. The two cohorts differed significantly in age, sex, race, education, and current smoking status. For the FDC exposure analyses, veterans who were exposed and unexposed did not differ on any of the demographic variables. However, adjusted analyses for exposure were performed to control for gender and current smoking, potential risk factors for these chronic conditions. Demographics are reported in Table 1.

### 3.2. Chronic Conditions in the FDC and NHANES Cohort

Non-restricted analyses are presented in Table 2. Non-restricted analyses revealed that FDC males had significantly higher odds of reporting HBP, high cholesterol, heart attack, arthritis, and chronic bronchitis than NHANES males. Restricted analyses revealed that the FDC males had significantly higher odds of reporting HBP, high cholesterol, heart attack, diabetes, stroke, arthritis, and chronic bronchitis (*p* < 0.05). The FDC excess prevalence ranged from 2.7 to 12.2% higher for these conditions (Table 2). This resulted in the FDC men having more than 2.5 times the odds of reporting a heart attack (OR = 2.63) and more than 4 times the odds of reporting chronic bronchitis (OR = 4.50). The odds of HBP, diabetes, and arthritis was at least 1.47 times higher in the FDC (Table 2).

**Figure 1:**
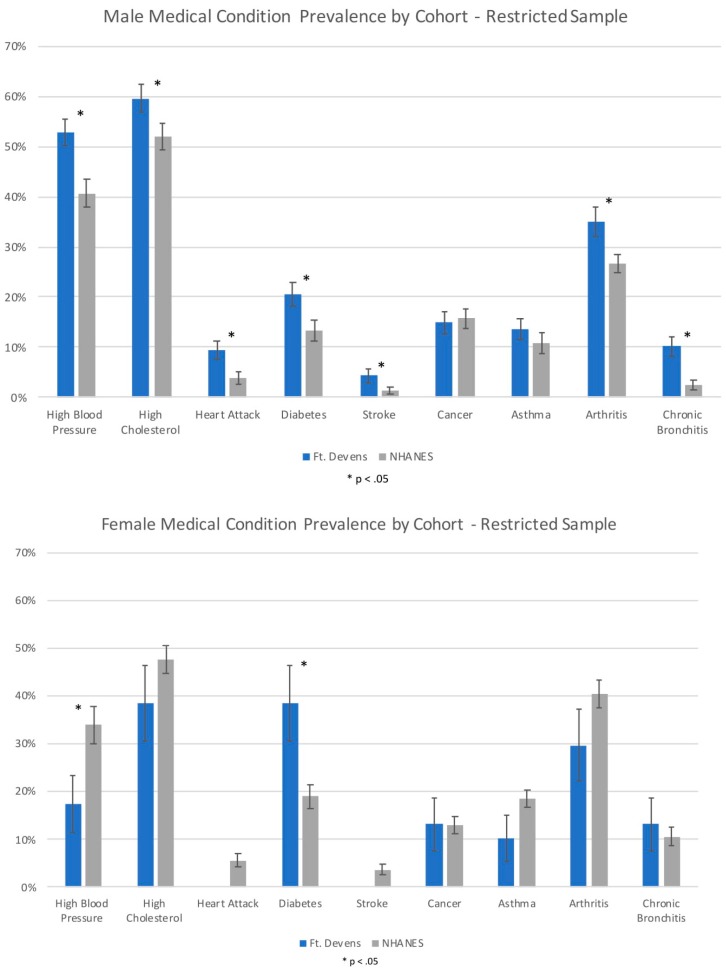
Prevalence of medical conditions in male and female participants from FDC and NHANES cohorts in the restricted sample. (Error bars represent Standard Error).

Non-restricted and restricted analyses revealed that the FDC females reported a significantly lower rate of HBP and a significantly higher rate of diabetes compared with the NHANES females (*p* < 0.05). The FDC women had a 16.4% lower prevalence of HBP and a 19.5% higher prevalence of diabetes compared to the NHANES general population. There was no significant association between cohort and high cholesterol, heart attack, stroke, cancer, asthma, arthritis, and chronic bronchitis (*p* > 0.05). FDC and NHANES cohort comparison results are shown in Figure 1 and Table 2.

### 3.3. Age and Gender Comparisons in FDC and NHANES Cohorts

For males in their 40s, the non-restricted analyses indicated significant associations between cohort and HBP and high cholesterol (*p* < 0.05), with the FDC reporting higher prevalence (Table 3). In the restricted analyses, there was a significant association between cohort and heart attack (*p* < 0.05), with the FDC reporting a higher prevalence than the NHANES cohort. The excess prevalence for heart attack was 4.4% (Table 3). The odds of FDC men reporting a heart attack was 27.36 times higher than NHANES men (Table 3). There was no significant association between cohort and the eight other chronic conditions.

For males in their 50s, the non-restricted analyses indicated significant associations between cohort and HBP, high cholesterol, cancer, arthritis and chronic bronchitis (*p* < 0.05), with the FDC reporting more chronic conditions. In the restricted analyses, there was a significant association between cohort and arthritis and chronic bronchitis (*p* < 0.05) with the FDC reporting more chronic conditions than NHANES men. The FDC excess prevalence for these conditions ranged from 9.0 to 17.9% (Table 3). The odds of FDC men reporting chronic bronchitis was 3.94 times higher than the NHANES men. There was no significant association between cohort and the seven other chronic conditions in this age group (*p* > 0.05).

For males in their 60s, the non-restricted analyses indicated significant associations between cohort and high cholesterol, stroke, cancer, asthma, arthritis, and chronic bronchitis (*p* < 0.05), with the FDC reporting higher prevalence. In the restricted analyses, there was a significant association between cohort and two chronic conditions including stroke and chronic bronchitis (*p* < 0.05) with FDC reporting higher rates than NHANES men. The excess prevalence was 12.1 and 10.5% respectively (Table 3). The odds of FDC men reporting stroke and chronic bronchitis were 5.69 and 4.83 times higher respectively than the NHANES men.

Non-restricted and restricted male age comparison results of prevalence and odds ratios are summarized in Table 3. Due to the small number of women in the FDC, female age grouping analyses did not yield sufficient power, and thus are not reported.

### 3.4. Chronic Conditions and Toxicant Exposures in the Ft. Devens Cohort

For the comparison of chronic medical conditions and toxicant exposures in the FDC, the same 9 chronic conditions were assessed as in the prior FDC and NHANES comparisons. There was a significant association between self-reported chemical/biological weapons (CBW) exposure and 4 chronic conditions (*p* < 0.05), with CBW-exposed veterans reporting higher prevalence rates of HBP, diabetes, arthritis and chronic bronchitis (Table 4). The excess prevalence of these conditions ranged from 10 to 21% higher prevalence. CBW-exposed veterans had greater than 4 times the odds of reporting diabetes in unadjusted analyses and 3 times the odds in adjusted analyses compared with unexposed veterans.

There was also a significant association between PB pill usage during the war and 2 chronic conditions (*p* < 0.05) with PB exposed veterans reporting higher prevalence rates of heart attack and diabetes (*p* < 0.05). The excess prevalence for these two conditions were 10% and 12.5% respectively. PB-exposed veterans had 11 times the odds of reporting a heart attack in unadjusted analyses and 12 times the odds in adjusted analyses than the non-PB-exposed FDC veterans (Table 4).

### 3.5. Direct Comparison of Prevalence of Chronic Conditions in Men and Women within the Ft. Devens Cohort

When rates of the 9 chronic conditions were directly compared between men and women in the FDC, gender was significantly associated with 3 outcomes: HBP, for which FDC men had nearly 3.5 times the odds of reporting compared to FDC women (OR = 3.45); high cholesterol, for which FDC men had more than 2 times the odds of reporting compared to FDC women (OR = 2.25); and diabetes, for which FDC men had decreased odds of reporting compared to FDC women (OR = 0.38). Gender was not associated with any of the other chronic conditions (Table 5).

## 4. Discussion

More than 25 years post-deployment, GW veterans continue to report more chronic health symptoms and chronic medical conditions than non-deployed veterans [1,4,9,10,12,13,14,22,23,30]. A recent meta-analysis of chronic health symptoms in deployed GW veterans versus non-deployed GW-era veterans showed that GW veterans had higher odds of reporting mood-cognition, fatigue, musculoskeletal, gastrointestinal, and dermatological symptoms compared with non-deployed veterans when 21 studies encompassing 129,000 veterans were compared [4]. The meta-analysis suggested that the symptom categories encompassed in the Kansas GWI criteria are appropriate for determining the multi-symptom disorder called GWI [1]. In addition to GWI, as veterans age, they continue to develop chronic medical conditions and diseases of aging, as would be expected in the general population. Chronic illness is common in aging individuals, and an estimate in 2000 reported that 45% of Americans had chronic conditions, with over 20% having multiple chronic conditions [31]. In general, non-veteran women report more increased chronic conditions than men and one report suggested that GW veteran women may show increased rates of chronic conditions that have been used as exclusionary medical conditions for the Kansas GWI criteria [32]. This raises the question of whether the exclusionary criteria currently used for GWI case criteria are still appropriate in an aging population of veterans, and whether these GW veterans are showing the same age-related rates in chronic health conditions as the general population, or are showing unique age- and gender-related patterns of development of chronic medical conditions. To our knowledge, the current study is the first to compare GW veterans’ health status to that of the general public by comparing the Ft. Devens Cohort (FDC) with the NHANES cohort and thus builds upon and extends prior GW studies comparing chronic health conditions.

Results showed that male GW veterans have significantly greater odds of developing chronic conditions including HBP, high cholesterol, heart attack, diabetes, stroke, arthritis, and chronic bronchitis. Specifically, FDC men in their 40s have 27 times higher odds of reporting a heart attack compared to the NHANES cohort of the same age. Rates of chronic conditions such as arthritis and chronic bronchitis also appear to be significantly higher in FDC males in their 50s and stroke risk is five times higher in the 60s age range compared to the NHANES cohort. These results correspond with prior accelerated aging cognitive patterns previously noted in the FDC veterans [27]. The fact that heart attack risk is significantly higher in the 4th decade, but not later in the 5–6th decades, in the FDC males suggests that GW veterans may be at increased risk for cardiovascular insults earlier than the general population. The additionally higher risk of stroke in the 6th decade of FDC males appears to suggest earlier cerebrovascular disease risk as well. These results also correspond with reports of increased prevalence over a 10-year period (1995–2005) for arthritis, hypertension, heart disease and diabetes in a large National Survey of Gulf War veterans [14]. Significant new onset of chronic conditions including hypertension, coronary heart disease, and arthritis was also reported by Li et al. (2011) in GW veterans when compared with non-deployed era veterans [14]. However, these studies did not specifically assess gender differences.

Important gender differences were also observed in this study. Male GW veterans had significantly higher odds of developing seven out of the nine chronic conditions assessed when compared with age-matched NHANES males. Conversely, female GW veterans had lower odds of developing HBP and higher odds of developing diabetes when compared with age-matched NHANES females. The FDC was one of the first studies to report a 1.8 times higher risk of GWI in women veterans [26]. Although there were trends for higher odds of developing medical conditions for women in this study, statistical differences were not found in this latest FDC survey, which showed that women veterans have at least the same rate of GWI than their male counterparts. In addition, prior studies in other GW veteran cohorts have reported increased chronic conditions and/or exclusionary medical conditions for GWI in women GW veterans [32]. The current study found fewer chronic conditions in women veterans compared with the general population, while the male veterans showed more chronic conditions than the general population sample of men. When FDC men and women were directly compared for rates of chronic conditions, men had higher rates of HBP and high cholesterol and lower rates of diabetes compared to FDC women. Therefore, it is clear that the trajectory of chronic conditions in male and female GW veterans is different and therefore, their treatment planning should be considered based on these different risk patterns [33,34]. Specifically, men should be aggressively screened and treated for HBP and high cholesterol and women should be screened for diabetes to reduce further potential heart disease and cardiovascular events.

GW veterans’ chronic medical conditions were also compared by exposure status. Results indicated that neurotoxicant exposures during deployment were associated with higher rates of chronic conditions, compared to unexposed FDC veterans. In several prior studies, CBWs including sarin and cyclosarin were associated with GWI, as well as with altered brain imaging outcomes and other chronic illnesses including brain cancer [19,20,35,36,37,38]. PB has been associated with increased risk of GWI in the FDC and other cohorts as well as with other health outcomes including symptom severity, cognitive decrements and altered neuroendocrine function [5,6,7,8,26,30,39,40]. Although prior studies have reported these chronic medical conditions to be higher in GW veterans, this is the first study that we are aware of to report these toxicant exposures to be related to increased prevalence of these chronic conditions.

Collectively, these results indicate that toxic wounds from GW deployment related exposures appear to be associated with more than the chronic health symptoms of GWI but also with chronic conditions of aging. These results are similar to those observed in aging Vietnam veterans who were also shown to have increased risk of developing HBP, diabetes and multiple types of cancer if they were exposed to Agent Orange, the signature toxic wound during the Vietnam era [25,41]. Further assessment of chronic conditions and their causes in other confirmatory cohorts of GW veterans and reconsideration of some Kansas GWI exclusionary criteria is warranted given the current findings. The chronic conditions identified in this study are of importance for future studies of GW veterans and the impact of potential accelerated aging and development of age-related neurodegenerative conditions. While the biological mechanisms for possible accelerated aging in GW veterans are unknown, recent studies in human research participants and laboratory animals have implicated mitochondrial dysfunction and chronic neuroinflammation in GWI [42,43,44,45]. Additionally, understanding prevalence rate differences or excess prevalence of these chronic conditions is important not only to devise appropriate inclusion and exclusion criteria for GWI in an aging population, but also to assess the impact of prior toxicant exposures on GWI pathology.

### Strengths and Limitations

The strengths of the current study are that it includes comparisons of chronic conditions in two population-based cohorts including a cohort of deployed GW veterans who were followed within a week after their return from the Gulf War and the well-designed general population cohort called the National Health and Nutrition Examination Surveys (NHANES). Both cohorts were designed as population-based survey study cohorts. The limitations of the current study are the relatively low response rate of the current FDC survey (35%), which could have introduced some response bias, where sicker veterans responded to the survey. However, recent other longitudinal cohorts of GW veterans including the National Survey of Gulf War veterans have reported almost identical response rates on recent surveys with no response bias when prior participant rates were compared suggesting this may be less of an issue with population-based GW veteran cohorts [24]. In addition, although the FDC men were reporting increased rates of chronic conditions compared to NHANES men, the FDC women veterans actually showed lower prevalence of most chronic conditions when compared with the NHANES cohort. These results suggest against response bias in that sicker GW women veterans were not in fact responding to the FDC cohort in this latest FDC survey. Additionally, the FDC includes a large number of National Guardsmen and Reservists, and thus may not be representative of all Gulf War veterans. Furthermore, the small number of women may have limited the analyses and added some uncertainty to the results. Due to demographic differences between cohorts, restricted analyses were performed that limited the sample to White/Caucasians with at least a high school education. This limits the generalizability of our findings to not only other Gulf War veterans but to Gulf War veterans of different gender, races, and education levels.

Lastly, in splitting the samples by age, the sample was smaller for each age group, which may have introduced statistical power issues. Future work is needed to replicate these findings with larger cohorts.

## 5. Conclusions

In summary, GW veterans in the FDC cohort had significantly increased odds for reporting chronic conditions including high blood pressure, high cholesterol, heart attack, diabetes, stroke, arthritis and chronic bronchitis compared with the NHANES general population cohort. This was true for comparison of males in the two cohorts with heart attack being higher in the FDC 4th decade and chronic bronchitis and arthritis being higher in the FDC 5th decade and the addition of stroke risk being higher is the 6th decade. Female comparisons showed a different pattern with FDC women veterans having significantly lower odds of high blood pressure and significantly higher odds of diabetes when compared with NHANES women. Direct comparisons of male and female FDC showed male veterans had higher odds for reporting HBP and high cholesterol and lower odds of reporting diabetes than FDC women veterans. Gulf War-related exposures, including self-report of chemical weapons, was associated with significantly higher odds of HBP, diabetes, arthritis and chronic bronchitis and self-report of PB exposure was associated with higher risk of heart attack and diabetes. Results indicate that GW veterans are at higher risk of chronic conditions than the general population and these risks are associated with self-reported toxicant exposures. These results should be considered when assessing the functionality of exclusionary criteria for currently used GWI case definitions.

## Figures and Tables

**Table 1:** Demographics.

Demographics	Ft. Devens (*n* = 448) Mean ± SD or N (%)	NHANES (*n* = 2959) Mean ± SD or N (%)
Age *	53.93 ± 8.33	55.86 ± 10.23
Male *	54.13 ± 8.38	54.74 ± 9.04
Female *	52.15 ± 7.75	56.68 ± 10.90
Sex *		
Male	401 (89.5)	1249 (42.3)
Female	47 (10.5)	1710 (57.7)
Race/Ethnicity *		
Black/African American	12 (2.7)	592 (10.4)
White/Caucasian	416 (92.9)	1189 (69.2)
Hispanic	7 (1.6)	731 (12.7)
Asian	1 (0.2)	389 (5.6)
Other/Multiracial	10 (2.2)	58 (1.9)
Missing	2 (0.4)	0 (0)
Education *		
Less than a high school diploma	1 (0.2)	762 (25.8)
High School Diploma	80 (17.9)	660 (22.3)
Other Training or Some College	171 (38.2)	803 (27.1)
College or Above	195 (43.5)	731 (24.7)
Missing	1 (0.2)	3 (0.1)
Current Smoking *		
Yes	73 (16.3)	588 (19.9)
No	372 (83.0)	2370 (80.1)
Missing	3 (0.7)	1 (0.0)
Exposures		
Self-reported Exposures		
Chemical/Biological Warfare (CBW)	178 (39.7)	
Missing CBW Data	131 (29.2)	
Pyridostigmine Bromide (PB) pills	247 (55.1)	
Missing PB Data	89 (19.9)	
Prevalence of Gulf War Illness (GWI)		
Fukuda Criteria	265 (59.2)	
Male	237 (59.1)	
Female	28 (59.6)	
Fukuda—Severe Criteria	58 (21.9)	
Male	52 (21.9)	
Female	6 (21.4)	
Kansas Criteria	147 (32.8)	
Male	132 (32.9)	
Female	15 (31.9)	
Prevalence of Chronic Fatigue Syndrome (CFS)	26 (9.4)	
Male	21 (8.7)	
Female	5 (13.5)	
Prevalence of Irritable Bowel Syndrome (IBS)	47 (16.4)	
Male	38 (15.4)	
Female	9 (23.1)	
Prevalence of Fibromyalgia	11 (3.7)	
Male	8 (3.1)	
Female	3 (7.7)	

* *p* < 0.05.

**Table 2:** Cohort comparison of prevalence of medical conditions—non-restricted sample.

Condition	Ft. Devens % (*n* = 401)	NHANES % (*n* = 1249)	Excess Prevalence [CI]	*p*-Value	OR [CI]
**Men**					
Self-reported doctor diagnosed medical conditions					
High Blood Pressure	52.9%	40.7%	12.3% [6.5–18.1]	0.000	1.64 [1.299–2.073]
High Cholesterol	59.5%	49.7%	9.8% [3.8–15.9]	0.001	1.49 [1.165–1.904]
Heart Attack	9.2%	4.7%	4.5% [0.6–8.4]	0.025	2.04 [1.093–3.805]
Diabetes	20.9%	15.5%	5.4% [−0.03–10.8]	0.051	1.44 [0.998–2.078]
Stroke	4.4%	2.3%	2.1% [−0.6–4.7]	0.133	1.93 [0.819–4.549]
Cancer	14.4%	12.3%	2.1% [−2.7–6.9]	0.391	1.20 [0.791–1.819]
Asthma	15.3%	10.4%	4.9% [−0.07–9.9]	0.053	1.56 [0.994–2.448]
Arthritis	35.9%	25.4%	10.5% [4.5–16.6]	0.001	1.65 [1.238–2.192]
Chronic Bronchitis	10.7%	2.6%	8.0% [4.1–11.9]	0.000	4.41 [2.138–9.078]
**Women** % (*n* = 47) % (*n* = 1710)					
Self-reported doctor diagnosed medical conditions					
High Blood Pressure	22.2%	46.8%	−24.6% [−37.3–(−11.8)]	0.000	0.32 [0.181–0.582]
High Cholesterol	37.2%	45.5%	−8.3% [−23.1–6.5]	0.271	0.71 [0.385–1.308]
Heart Attack	2.5%	4.5%	−2.0% [−7.0–3.1]	0.440	0.55 [0.117–2.541]
Diabetes	37.2%	17.4%	19.8% [5.1–34.4]	0.008	2.81 [1.308–6.035]
Stroke	0%	4.1%	−4.1% [−5.1–(−3.1)]	N/A	N/A
Cancer	12.2%	12.8%	−0.6% [−10.8–9.5]	0.904	0.94 [0.373–2.387]
Asthma	9.5%	18.1%	−8.6% [−17.8–0.6]	0.067	0.48 [0.215–1.054]
Arthritis	29.3%	41.2%	−11.9% [−26.2–2.4]	0.104	0.59 [0.314–1.113]
Chronic Bronchitis	17.1%	9.6%	7.4% [−4.3–19.1]	0.213	1.93 [0.686–5.424]
**Men – Restricted Analyses**	% (*n* = 372)	% (*n* = 373)			
Self-reported doctor diagnosed medical conditions					
High Blood Pressure	52.7%	40.6%	12.2% [4.7–19.6]	0.001	1.63 [1.208–2.211]
High Cholesterol	59.6%	51.9%	7.7% [0.2–15.2]	0.044	1.37 [1.009–1.855]
Heart Attack	9.4%	3.8%	5.6% [1.3–9.9]	0.010	2.63 [1.255–5.530]
Diabetes	20.5%	13.4%	7.1% [0.9–13.4]	0.025	1.67 [1.066–2.620]
Stroke	4.3%	1.4%	2.9% [0.1–5.6]	0.041	3.19 [1.050–9.679]
Cancer	14.9%	15.7%	−0.8% [−6.6–5.0]	0.782	0.94 [0.602–1.465]
Asthma	13.6%	10.9%	2.7% [−3.1–8.6]	0.362	1.29 [0.746–2.228]
Arthritis	35.0%	26.7%	8.2% [1.5–15.0]	0.017	1.47 [1.073–2.026]
Chronic Bronchitis	10.2%	2.5%	7.7% [3.6–11.8]	0.000	4.50 [2.017–10.029]
**Women – Restricted Analyses**	% (*n* = 42)	% (*n* = 639)			
Self-reported doctor diagnosed medical conditions					
High Blood Pressure	17.5%	33.9%	−16.4% [−30.3–(−2.5)]	0.021	0.41 [0.196–0.874]
High Cholesterol	38.5%	47.7%	−9.2% [−25.6–7.0]	0.266	0.68 [0.351–1.334]
Heart Attack	0%	5.6%	−5.6% [−8.4–(−2.8)]	N/A	N/A
Diabetes	38.5%	19.0%	19.5% [3.4–35.6]	0.017	2.67 [1.189–6.001]
Stroke	0%	3.7%	−3.7% [−5.9–(−1.5)]	N/A	N/A
Cancer	13.2%	13.0%	0.2% [−11.1–11.4]	0.977	1.01 [0.377–2.733]
Asthma	10.3%	18.6%	−8.3% [−18.5–1.8]	0.109	0.50 [0.215–1.166]
Arthritis	29.7%	40.4%	−10.6% [−26.4–5.1]	0.187	0.63 [0.312–1.255]
Chronic Bronchitis	13.2%	10.6%	2.5% [−8.9–14.0]	0.663	1.28 [0.427–3.820]

* Actual N of each medical conditions may vary due to missing data.

**Table 3:** Cohort comparison of male rates of medical conditions by age group.

Condition	Ft. Devens	NHANES	Excess Prevalence [CI]	*p*-Value	OR [CI]
**40s**	% (*n* = 142)	% (*n* = 387)			
Self-reported doctor diagnosed medical conditions					
High Blood Pressure	43.6%	26.4%	17.2% [7.9–26.5]	0.000	2.16 [1.423–3.272]
High Cholesterol	50.8%	37.6%	13.2% [3.3–23.1]	0.009	1.71[1.144–2.561]
Heart Attack	4.5%	1.8%	2.7% [−1.4–6.7]	0.199	2.54 [0.612–10.509]
Diabetes	14.2%	9.3%	4.9% [−2.2–12.0]	0.174	1.62 [0.808–3.232]
Stroke	0%	0.8%	−0.8% [−1.6–0.01]	N/A	N/A
Cancer	5.4%	2.8%	2.6% [−2.0–7.1]	0.267	1.95 [0.599–6.373]
Asthma	12.8%	10.9%	2.0% [−5.0–9.0]	0.578	1.21 [0.618–2.373]
Arthritis	17.0%	12.2%	4.8% [−3.1–12.7]	0.231	1.48 [0.780–2.789]
Chronic Bronchitis	4.7%	1.8%	2.9% [−1.4–7.1]	0.183	2.66 [0.629–11.256]
**50s**	% (*n* = 149)	% (*n* = 382)			
High Blood Pressure	55.1%	40.8%	14.3% [4.8–23.7]	0.003	1.78 [1.215–2.602]
High Cholesterol	61.3%	45.8%	15.5% [5.6–25.4]	0.002	1.87 [1.253–2.805]
Heart Attack	9.3%	4.2%	5.1% [−1.0–11.2]	0.103	2.34 [0.842–6.503]
Diabetes	20.0%	15.2%	4.8% [−3.7–13.3]	0.270	1.39 [0.773–2.509]
Stroke	2.1%	2.6%	−0.5% [−3.8–2.7]	0.746	0.79 [0.188–3.311]
Cancer	17.6%	6.5%	11.1% [3.3–18.9]	0.005	3.06 [1.393–6.723]
Asthma	13.4%	10.2%	3.2% [−4.3–10.6]	0.404	1.36 [0.662–2.782]
Arthritis	43.8%	22.0%	21.8% [11.4–32.1]	0.000	2.76 [1.700–4.471]
Chronic Bronchitis	13.1%	2.6%	10.5% [3.7–17.4]	0.003	5.62 [1.824–17.335]
**60s**	% (*n* = 96)	% (*n* = 480)			
High Blood Pressure	67.4%	58.6%	8.8% [−1.6–19.2]	0.098	1.46 [0.932–2.286]
High Cholesterol	71.1%	54.4%	16.7% [5.9–27.4]	0.002	2.06 [1.292–3.279]
Heart Attack	20.3%	10.2%	10.1% [−0.5–20.8]	0.062	2.25 [0.960–5.252]
Diabetes	34.4%	29.9%	4.4% [−8.0–16.8]	0.483	1.23 [0.694–2.166]
Stroke	16.1%	5.8%	10.2% [0.4–20.1]	0.042	3.08 [1.041–9.139]
Cancer	27.6%	15.2%	12.4% [0.4–24.3]	0.043	2.12 [1.026–4.398]
Asthma	23.2%	9.8%	13.4% [2.0–24.8]	0.021	2.79 [1.168–6.643]
Arthritis	55.0%	34.5%	20.5% [7.2–33.8]	0.003	2.32 [1.342–4.004]
Chronic Bronchitis	15.1%	3.8%	11.3% [1.5–21.1]	0.024	4.54 [1.226–16.837]
**40s – Restricted Analyses**	% (*n* = 134)	% (*n* = 124)			
High Blood Pressure	42.4%	29.0%	13.4% [−0.2–27.0]	0.054	1.80 [0.991–3.279]
High Cholesterol	50.0%	47.3%	2.7% [−11.1–16.5]	0.704	1.11 [0.641–1.933]
Heart Attack	4.6%	0.2%	4.4% [0.5–8.4]	0.029	27.36 [1.413–529.776]
Diabetes	13.8%	7.2%	6.6% [−2.9–16.1]	0.175	2.06 [0.725–5.867]
Stroke	0%	0%	0%	N/A	N/A
Cancer	5.6%	6.7%	−1.2% [−8.0–5.7]	0.741	0.82 [0.246–2.709]
Asthma	11.4%	11.0%	0.4% [−7.9–8.7]	0.918	1.04 [0.454–2.405]
Arthritis	16.3%	19.0%	−2.7% [−13.5–8.2]	0.629	0.83 [0.394–1.757]
Chronic Bronchitis	4.8%	0.9%	3.9% [−0.4–8.2]	0.077	5.51 [0.829–36.651]
**50s – Restricted Analyses**	% (*n* = 135)	% (*n* = 123)			
High Blood Pressure	56.4%	43.1%	13.3% [−2.7–29.4]	0.104	1.71 [0.896–3.259]
High Cholesterol	63.1%	49.9%	13.2% [−0.1–26.4]	0.051	1.71 [0.997–2.947]
Heart Attack	10.5%	3.6%	6.9% [−0.8–14.6]	0.080	3.14 [0.871–11.340]
Diabetes	20.2%	14.4%	5.9% [−5.1–16.8]	0.295	1.51 [0.698–3.272]
Stroke	2.4%	1.7%	0.7% [−3.3–4.7]	0.732	1.43 [0.184–11.088]
Cancer	18.7%	17.0%	1.7% [−10.4–13.8]	0.788	1.12 [0.490–2.559]
Asthma	11.6%	10.2%	1.4% [−7.6–10.5]	0.755	1.16 [0.458–2.934]
Arthritis	43.0%	25.1%	17.9% [5.9–29.9]	0.004	2.25 [1.306–3.876]
Chronic Bronchitis	12.5%	3.5%	9.0% [1.4–16.6]	0.020	3.94 [1.242–12.507]
**60s+ - Restricted Analyses**	% (*n* = 89)	% (*n* = 126)			
High Blood Pressure	67.0%	54.2%	12.9% [−1.5–27.2]	0.079	1.72 [0.939–3.153]
High Cholesterol	70.5%	62.0%	8.5% [−6.6–23.6]	0.269	1.47 [0.744–2.892]
Heart Attack	19.6%	9.6%	10.0% [−2.3–22.4]	0.112	2.30 [0.824–6.441]
Diabetes	32.8%	21.4%	11.4% [−2.2–25.0]	0.100	1.79 [0.894–3.597]
Stroke	15.1%	3.0%	12.1% [1.9–22.2]	0.020	5.69 [1.313–24.695]
Cancer	28.6%	27.4%	1.2% [−13.7–16.0]	0.877	1.06 [0.507–2.215]
Asthma	20.8%	11.8%	8.9% [−3.8–21.7]	0.170	1.96 [0.750–5.101]
Arthritis	55.4%	40.7%	14.6% [−0.9–30.2]	0.065	1.80 [0.963–3.379]
Chronic Bronchitis	13.7%	3.2%	10.5% [0.6–20.5]	0.038	4.83 [1.092–22.359]

* Actual N may vary due to missing data.

**Table 4:** Rates of medical conditions for exposed and unexposed Ft. Devens veterans.

Condition	Exposed % (*n* = 178)	Unexposed % (*n* = 139)	Excess Prevalence	*p*-Value	OR [CI]	Adjusted OR [CI]
**Chemical/Biological Warfare (CBW)**						
Self reported doctor diagnosed medical conditions						
High Blood Pressure	54.0%	42.0%	12.0%	0.040	1.621 [1.033–2.544]	1.601 [1.012–2.533]
High Cholesterol	56.8%	53.4%	3.4%	0.633	1.145 [0.717–1.827]	1.084 [0.674–1.742]
Heart Attack	11.0%	5.5%	5.5%	0.161	2.147 [0.796–5.795]	2.013 [0.742–5.464]
Diabetes	29.9%	8.9%	21.0%	0.000	4.340 [2.055–9.165]	3.242 [1.647–6.382]
Stroke	4.1%	3.7%	0.4%	1.000	1.122 [0.293–4.288]	1.116 [0.289–4.310]
Cancer	17.5%	11.4%	6.1%	0.204	1.643 [0.785–3.439]	1.494 [0.722–3.090]
Asthma	18.5%	10.3%	8.2%	0.094	1.987 [0.919–4.296]	1.940 [0.896–4.201]
Arthritis	43.4%	23.9%	19.5%	0.002	2.446 [1.403–4.265]	2.199 [1.261–3.834]
Chronic Bronchitis	16.0%	5.7%	10.3%	0.020	3.175 [1.225–8.229]	3.997 [1.426–11.202]
Chronic Fatigue Syndrome (CFS)	15.2%	2.8%	12.4%	0.002	6.131 [1.717–21.889]	5.957 [1.664–21.321]
Irritable Bowel Syndrome (IBS)	24.8%	8.7%	16.1%	0.003	3.439 [1.529–7.734]	3.401 [1.502–7.699]
**Pyridostigmine Bromide (PB) Pills** % (*n* = 247) % (*n* = 112)						
Self reported doctor diagnosed medical conditions						
High Blood Pressure	50.2%	40.9%	9.3%	0.109	1.456 [0.923–2.299]	1.542 [0.969–2.453]
High Cholesterol	58.4%	51.0%	7.4%	0.226	1.351 [0.840–2.174]	1.305 [0.807–2.110]
Heart Attack	11.5%	1.1%	10.4%	0.003	11.348 [1.501–85.798]	12.141 [1.601–92.054]
Diabetes	23.7%	11.2%	12.5%	0.015	2.452 [1.172–5.128]	2.175 [1.107–4.273]
Stroke	4.6%	1.1%	3.5%	0.280	4.193 [0.516–34.067]	4.673 [0.572–38.192]
Cancer	15.3%	10.1%	5.2%	0.265	1.606 [0.723–3.567]	1.443 [0.666–3.124]
Asthma	12.5%	11.5%	1.0%	0.845	1.100 [0.496–2.438]	1.127 [0.507–2.505]
Arthritis	36.4%	31.4%	5.0%	0.494	1.249 [0.725–2.152]	1.223 [0.711–2.105]
Chronic Bronchitis	13.1%	8.0%	5.1%	0.227	1.751 [0.720–4.255]	1.664 [0.678–4.085]
Chronic Fatigue Syndrome (CFS)	10.9%	2.4%	8.5%	0.037	4.885 [1.094–21.809]	5.076 [1.132–22.760]
Irritable Bowel Syndrome (IBS)	20.9%	8.6%	12.3%	0.017	2.791 [1.175–6.627]	2.662 [1.112–6.372]

* Actual N of each medical condition may vary due to missing data. Adjusted ORs control for Gender and Current Smoking.

**Table 5:** Direct comparison of medical condition prevalence in men & women of the Ft. Devens cohort.

Medical Condition	Men	Women	*p*-Value	OR [CI]	* Adjusted OR [CI]
High Blood Pressure	52.9%	22.2%	0.000	3.933 [1.895–1.895]	3.454 [1.622–7.353]
High Cholesterol	59.5%	37.2%	0.006	2.484 [1.291–4.777]	2.249 [1.127–4.488]
Heart Attack	9.2%	2.5%	0.224	3.961 [0.523–30.024]	3.253 [0.415–25.466]
Diabetes	20.9%	37.2%	0.021	0.447 [0.227–0.881]	0.378 [0.182–0.782]
Stroke	4.4%	0.0%	0.246	N/A	N/A
Cancer	14.4%	12.2%	0.815	1.215 [0.450–3.277]	1.099 [0.390–3.100]
Asthma	15.3%	9.5%	0.363	1.712 [0.581–5.050]	1.570 [0.527–4.679]
Arthritis	35.9%	29.3%	0.485	1.354 [0.662–2.769]	1.296 [0.592–2.835]
Chronic Bronchitis	10.7%	17.1%	0.289	0.580 [0.236–1.426]	0.460 [0.179–1.182]

* Adjusted ORs control for Age, Race, Education, and Current Smoking.
